# A Novel Approach to Assessment of US Pediatric Trauma System Development

**DOI:** 10.1001/jamasurg.2022.4303

**Published:** 2022-09-21

**Authors:** Mary E. Fallat, Colin Treager, Sophie Humphrey, Lindsey Gumer, Kahir Jawad, Elissa Butler, Frederick B. Rogers, Frederick P. Rivara, Amelia T. Collings

**Affiliations:** 1 Norton Children’s Hospital, Louisville, Kentucky; 2 Hiram C. Polk, Jr Department of Surgery, University of Louisville School of Medicine, Louisville, Kentucky; 3 Norton Children’s Research Institute, Louisville, Kentucky; 4 University of Texas at Austin, Dell Medical School, Austin; 5 University of Kentucky School of Medicine, Lexington; 6 IQVIA, Louisville, Kentucky; 7 State University of New York, Upstate Medical University, Syracuse; 8 Department of Surgery, Regions Hospital, St Paul, Minnesota; 9 Department of Pediatrics and the Harborview Injury Prevention and Research Center, University of Washington, Seattle

## Abstract

**Importance:**

Mature trauma systems are critical in building and maintaining national, state, and local resilience against all-hazard disasters. Currently, pediatric state trauma system plans are not standardized and thus are without concrete measures of potential effectiveness.

**Objective:**

To develop objective measures of pediatric trauma system capability at the state level, hypothesizing significant variation in capabilities between states, and to provide a contemporary report on the status of national pediatric trauma system planning and development.

**Design, Setting, and Participants:**

A national survey was deployed in 2018 to perform a gap analysis of state pediatric trauma system capabilities. Four officials from each state were asked to complete the survey regarding extensive pediatric-related or specific trauma system parameters. Using these parameters, a panel of 14 individuals representing national stakeholder sectors in pediatric trauma care convened to identify the essential components of the ideal pediatric trauma system using Delphi methodology. Data analysis was conducted from March 16, 2019, to February 23, 2020.

**Main Outcomes and Measures:**

Based on results from the national survey and consensus panel parameters, each state was given a composite score. The score was validated using US Centers for Disease Control and Prevention Wide-Ranging Online Data for Epidemiologic Research (CDC WONDER) fatal injury database.

**Results:**

The national survey had less than 10% missing data. The consensus panel reached agreement on 6 major domains of pediatric trauma systems (disaster, legislation/funding, access to care, injury prevention/recognition, quality improvement, pediatric readiness) and was used to develop the Pediatric Trauma System Assessment Score (PTSAS) based on 100 points. There was substantial variation across states, with state scores ranging from 48.5 to 100. Based on US CDC WONDER data, for every 1-point increase in PTSAS, there was a 0.12 per 100 000 decrease in mortality (95% CI, −0.22 to −0.02; *P* = .03).

**Conclusions and Relevance:**

Results of this cross-sectional study suggest that a more robust pediatric trauma system has a significant association with pediatric injury mortality. This study assessed the national landscape of capability and preparedness to provide pediatric trauma care at the state level. These parameters can tailor the maturation of children’s interests within a state trauma system and assist with future state, regional, and national planning.

## Key Points

**Question:**

What is the current landscape of state pediatric trauma system development in the US?

**Findings:**

After performing a cross-sectional study of each state’s pediatric trauma capabilities, an expert panel developed an objective assessment of state pediatric trauma systems using Delphi methodology. The Pediatric Trauma System Assessment Score (PTSAS) was externally validated, showing that a more mature state trauma system significantly decreased child mortality from injury.

**Meaning:**

PTSAS can be used to tailor a state trauma system to children’s interests and assist with future state, regional, and national planning.

## Teaser

This cross-sectional study investigates state pediatric trauma system development in the US.

## Introduction

Injury is a leading cause of death in the US and the most common cause of death in children and adults up to age 44 years. The threat is magnified when considering the increasing frequency of unexpected natural and man-made incidents. High-functioning trauma systems play a vital role in building and maintaining national, state, local, and tribal resilience against these disasters. Currently, pediatric state trauma plans are not standardized and are without concrete measures of potential effectiveness.

The historic and guiding principles for trauma system development using the public health approach are embedded within the 2004 Trauma System Agenda for the Future.^1^ The Health Resources & Services Administration (HRSA) 2006 Model Trauma System Planning and Evaluation (MTSPE) document served as the basis for US trauma system development and is foundational for the American College of Surgeons Committee on Trauma (ACS-COT) Trauma Systems Consultation program.^2^ The MTSPE included an assessment tool, comprising a series of Benchmarks, Indicators, and Scoring (BIS) criteria. The current MTPSE and BIS scoring tools do not consider pediatric issues within a trauma system.

Trauma involves a continuum of care, beginning with injury prevention and prehospital care, progressing through emergency department, intensive, and acute care, and ending with rehabilitation and community reintegration. A 2016 report from the National Academies of Sciences, Engineering, and Medicine confirms the need for stronger integration, particularly the need to integrate military and civilian trauma systems, as well as prehospital and trauma center care.^3^ Current issues in trauma system development include limited financial support for infrastructure, the need for expansion of disaster preparedness programs and improved data systems, and strategies for system-wide quality improvement.

A report issued in 2016 by the General Accountability Office (GAO) commissioned by a bipartisan congressional pediatric trauma caucus described location of children in proximity to state or ACS-COT designated trauma centers from 2011 to 2015.^4^ The National Association of State Emergency Medical Services Officials (NASEMSO) released an updated monograph in 2017 that provided a snapshot of the status of state trauma system development using the system development tools provided in the MTSPE.^5^ The NASEMSO report made 2 relevant points: (1) formal trauma systems do not exist in all states and (2) the standards, criteria, and requirements that guide state trauma systems are not directly comparable, owing to differing definitions of terms, inclusion and exclusion criteria for data systems, and processes for recognition of trauma centers.^6^ This report provided minimal information on pediatric resources within existing state trauma systems.

The current study includes a gap analysis to inform trauma system development parameters that include children’s interests. Our primary aim was to develop objective measures of pediatric trauma system capability at the state level, hypothesizing significant variation in capabilities between states. Our secondary aims included to (1) provide a contemporary report on the status of national pediatric trauma system planning and development, (2) develop a novel scoring system to evaluate the maturity of pediatric trauma systems, and (3) evaluate the utility of this scoring system in predicting pediatric trauma outcomes at the state level.

## Methods

### State Survey

The institutional review board of the University of Louisville approved this study and provided a waiver for participant informed consent owing to the use of deidentified data. Baseline data from the 2016 NASEMSO report on state utilization of HRSA’s MTSPE, last updated in 2006 and used by the ACS-COT to evaluate trauma systems and a 2016 US GAO report on the status of pediatric trauma centers,^4,5^ were abstracted by state in a spreadsheet. An online survey (eTable 1 in the Supplement) was developed and distributed via Survey Monkey (Momentive) to perform a cross-sectional gap analysis and was sent in February 2018 to 4 state officials: the EMS director, EMSC program manager, trauma program manager, and ACS-COT chair. Each state was also sent the spreadsheet with their data from the 2 existing reports to verify if this information was still correct and were given an opportunity to update the information. This study followed the Strengthening the Reporting of Observational Studies in Epidemiology (STROBE) reporting guidelines.

The survey included questions related to pediatric representation in state trauma advisory leadership, trauma center designation, trauma triage guidelines, a pediatric trauma registry, and integration of children into the disaster plan. The survey was developed with input from stakeholders including members of the American Pediatric Surgical Association, ACS-COT, and NASEMSO. The final survey received input from the ACS psychometrician.

The 4 state officials were asked to work together with 1 person taking the lead to compile the results and enter them into the online survey platform. State results were integrated with GAO and NASEMSO trauma system reports to compile an overview of the landscape of pediatric trauma systems across the country. Compiled results were redistributed to each group to confirm accuracy.

If the group of 4 state officials answered the questions independently, differently, and did not work as a team resulting in conflicting answers, the investigator group worked to reconcile the differences before sending the survey back to the team for validation. Some state positions were not filled at the time of the first request. There were several states where no one responded, and we reached out to key organizations to assist with finding contacts (eFigure 1 in the Supplement). Some state officials retired or left their positions between when the survey was deployed the first time and when it was resent for validation, and an entirely new state official was examining the abstracted data for the first time. Conflicting data were reconciled to the best of our ability, and missing data were filled in using resources and public documents on the internet, primarily in 2020 to 2021 (eFigure 1 in the Supplement).

### Pediatric Trauma System Assessment Score Development

To develop a state-level score of pediatric trauma system capability, called the Pediatric Trauma System Assessment Score (PTSAS), a panel of 15 experts in pediatric trauma were selected to be part of a Delphi group.^7^ The panelists’ expertise spanned the continuum of trauma care (eTable 2 in the Supplement). The initial step was to identify seminal parameters of pediatric trauma capacity within a state’s trauma system. Delphi participants were presented with 30 different parameters, chosen from the survey questions most relevant to state pediatric trauma system development. Panel members were asked to score each from 1 to 10 based on relative importance, with 1 being least important and 10 being most important. They were given the opportunity to explain their answers and show evidence for their responses. All survey responses were anonymous. During subsequent rounds of surveys, the parameters were ranked based on the weighted average from the previous round. In addition, the experts were given the anonymous responses of their fellow panelists, with the goal being eventual consensus. The scoring system underwent a total of 5 survey rounds. Fourteen members completed the first 3 surveys, and 13 members completed the last 2 surveys.

Based on the results of the first 2 rounds of the Delphi scoring, the working group eliminated parameters that had a weighted average below 7.0 and/or combined related parameters to reduce redundancy. There was unanimous agreement on the importance of adding a parameter to describe state involvement in the National Pediatric Readiness Project (NPRP), which defines pediatric readiness in hospitals as ensuring that every emergency department has the right equipment, supplies, medications, and training to provide high-quality emergency care for children.^8^ After completing an assessment based on a checklist, the NPRP respondents receive a gap report, which highlights areas of competence and quality improvement opportunities for their own emergency department. The NPRP gap report provides a pediatric readiness score out of a possible 100 points that can be compared with the national average. For purposes of the PTSAS, the parameter “the state measures pediatric readiness in its emergency departments” was defined as 80% or greater participation in the 2011 survey that was reported in 2013 (representing the data available for review during the period of study) by a state’s emergency departments.

The panel agreed on 6 domains (injury prevention/recognition, access to care, pediatric readiness, quality improvement, disaster, legislation/funding). Each domain was scored on the 1 to 10 scale and averaged across panel members’ scores in the final round of Delphi voting. A PTSAS summary score ranging from 0 to 100 was computed based on the weighted averages of each domain and points distributed to the 3 to 5 capability parameters within those domains. Every parameter was dichotomous, and if present, the state received the full weighted value for that parameter. Each state was assigned a PTSAS based on state officials’ responses and GAO and NASEMSO trauma system reports. Results by region were created based on the various regionalization schemas of the organizations that contributed to the development of this study (Table 1).

**Table 1.** Regionalization Schemas Used for the Creation of Pivot Tables

Organization	Abbreviation	No. of regions	Rationale for inclusion
Emergency Medical Services for Children	EMSC	9	HRSA-funded program for EMS and emergency department preparedness for children’s emergencies
American College of Surgeons Committee of Trauma	ACS-COT	10	Regionalization for state trauma system development
National Association of State EMS Officials	NASEMSO	5	This organization houses the state trauma directors and has provided 2 historic reports of state trauma system development, including 1 used in our study
US Census Bureau	NA	9	Used the WONDER database for PTSAS validation
American Burn Association	ABA	5	Burn care is an integral part of trauma care, but the regions are different

Abbreviations: EMS, Emergency Medical Services; HRSA, Health Resources & Services Administration; NA, not applicable; PTSAS, Pediatric Trauma System Assessment Score; WONDER, Wide-Ranging Online Data for Epidemiologic Research.

### PTSAS Validation

To validate the PTSAS, we assessed the correlation of PTSAS with state pediatric injury mortality. The US Centers for Disease Control and Prevention Wide-Ranging Online Data for Epidemiologic Research (CDC WONDER) underlying cause of death database is an online database that provides mortality and population counts by state and cause of death based on death certificates.^9^ We queried the database for the number and rate of death owing to injury in children aged 0 to 17 years in each state for 2016 to 2017 (based on the CDC WONDER External Cause of Injury Mortality Matrix for *International Statistical Classification of Diseases and Related Health Problems*,* Tenth Revision*, all intents and mechanisms). These years were chosen to best reflect the status of pediatric trauma systems at the time of the NASEMSO and GAO reports and our survey in February 2018.

### Statistical Analysis

The overall mortality rate was calculated and was further stratified by place of death (in-hospital and out of hospital). In-hospital death was defined as those who died in a medical facility, which included inpatient, outpatient or emergency department, dead on arrival, and medical facility (specifics unknown). Out-of-hospital death was defined as the decedent’s home, hospice facility, nursing home or long-term care facility, and other, which includes trauma scene deaths. We used a linear regression to determine the association between PTSAS and pediatric injury mortality rates (overall, in-hospital, and out of hospital) at the state level. Data preparation and statistical analyses were performed with SAS software, version 9.4 (SAS Institute) and R software, version 4.1.1 (R Foundation). Statistical significance was defined as a 2-sided *P* value < .05. Data analysis was conducted from March 16, 2019, to February 23, 2020.

## Results

The data for the state surveys were compiled from state officials (2283 of 4226 [54%]), legacy data (1299 of 4226 [31%]), which included data from US Census Bureau, NASEMSO’s 2016 Report, and the GAO report on pediatric trauma. The study team was able to supplement 6% of parameters (257 of 4226) from publicly available documents. A total of 387 of 4226 (9%) of data was unfilled by state officials and unable to be found in public documents, and therefore was considered missing. The only parameter that was fulfilled by all 50 states and Washington DC was “mass casualty drills include both a process for identifying children to be moved and verifying facilities receiving children as having appropriate resources to provide optimal care.” In contrast, the parameter with the lowest compliance was “there are state funds designated for pediatric trauma care.” Only 39% states (20 of 51) reported having funds available for pediatric trauma care. Individual parameter compliance is reported in Table 2. State-level data for each PTSAS parameter are available in eTable 4 in the Supplement.

**Table 2.** Pediatric Trauma System Assessment Score (PTSAS) Domains and Parameters, Including State Compliance

Domain	Weighted average	Parameter	PTSAS points (total = 100)	States in compliance, No. (%)
Disaster	16.99	State disaster plan includes children	4.55	36 (71)
		State holds mass casualty drills that include children	3.87	44 (86)
		Mass casualty drills include facilities planning for transfer of children to accommodate surge	4.36	30 (59)
		Mass casualty drills include both a process for identifying children to be moved and verifying facilities receiving children as having appropriate resources to provide optimal care	4.21	51 (100)
Legislation and funding	15.79	There is state legislation for trauma system development	3.43	49 (96)
		There is mandatory pediatric representation on your state Trauma Advisory Council	3.38	34 (67)
		State trauma legislation specifically addresses injured children and includes planning, simulation, and modeling	3.14	32 (63)
		State has enabling legislation to designate pediatric trauma centers	2.95	33 (65)
		There are state funds designated for pediatric trauma care	2.89	20 (39)
Access to care	17.59	State has an EMS patient triage or destination determination protocol (eg, Guidelines for Field Triage of Injured Patients) for injured children (nearest hospital vs appropriate trauma center)	3.99	25 (49)
		State has access to inpatient rehabilitation beds available for children under 14 y old in a pediatric rehabilitation unit (the unit can be within a rehabilitation facility but is specifically designated for children)^a^	3.35	47 (92)
		State has access to burn beds available for children^a^	3.58	41 (80)
		Majority (>50%) of pediatric patients that live within 30 mi of either a high-level (I or II) pediatric or adult trauma center	3.08	45 (90)
		Majority (>50%) of pediatric patients that live within 30 mi of either a high- or mid-level (I, II, or III) pediatric or adult trauma center	3.58	50 (98)
Injury Prevention and Recognition	15.65	State legislation is in place to review all child fatalities due to injury, including child abuse	5.30	41 (80)
		All levels of trauma center (adult, pediatric, or mixed) have education programs for their staff that include recognition of child abuse	5.34	43 (84)
		State agencies, health department, or the trauma system lead efforts in organized injury prevention for children	5.01	50 (98)
Quality Improvement and Trauma Registry	16.99	Summary data from state-based trauma registry is publicly reported and includes pediatric trauma patients	5.75	33 (65)
		Trauma registry data in the state is used for children's performance improvement and is evaluated separately from adults	5.91	29 (57)
		State pediatric EMS data are used for EMS service or system PI and are evaluated separately from adults	5.33	34 (67)
Pediatric readiness	16.99	The state measures pediatric readiness of its emergency departments	6.08	35 (69)
		State requires transfer guidelines and defined processes/protocols be in place at each hospital	5.77	36 (71)
		Hospitals in the state, in general, use as low as reasonably achievable (ALARA) guidelines for radiographic imaging	5.15	36 (71)

Abbreviations: EMS, Emergency Medical Services; PI, performance improvement.

^a^ Resources may be available within state borders or at burn or rehabilitation centers in neighboring states.

**Figure 1.**
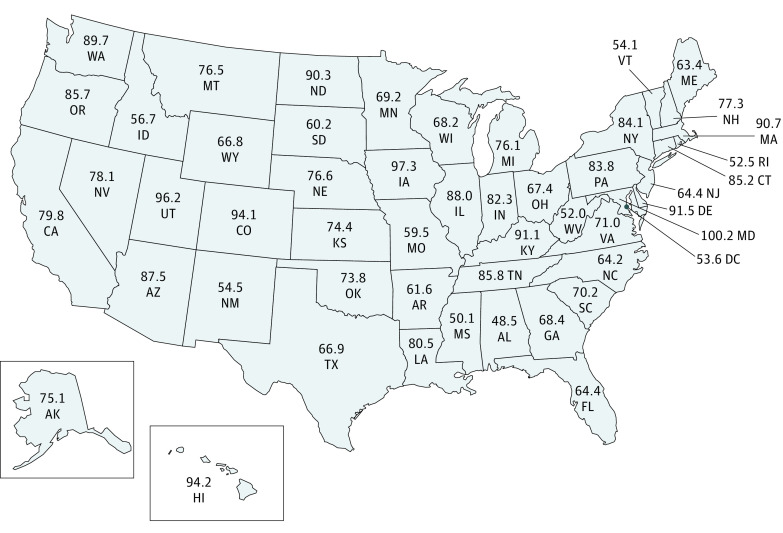
Map of States With Their Pediatric Trauma System Assessment Score

Each state was assigned a PTSAS (Figure 1). The scoring system underwent a total of 5 survey rounds (eTable 3 in the Supplement). The mean (SD) national PTSAS was 74.4 (14.1). Alabama had the lowest score at 48.5, whereas Maryland had the highest at 100. In 2016-2017, the annual national injury mortality rate in children was 14.2 per 100 000, which also included place of death category “unknown,” but not included in the in-hospital and out-of-hospital mortality. When classified by place of death, in-hospital mortality was 7.4 per 100 000, and out-of-hospital mortality was 3.3 per 100 000.

**Figure 2.**
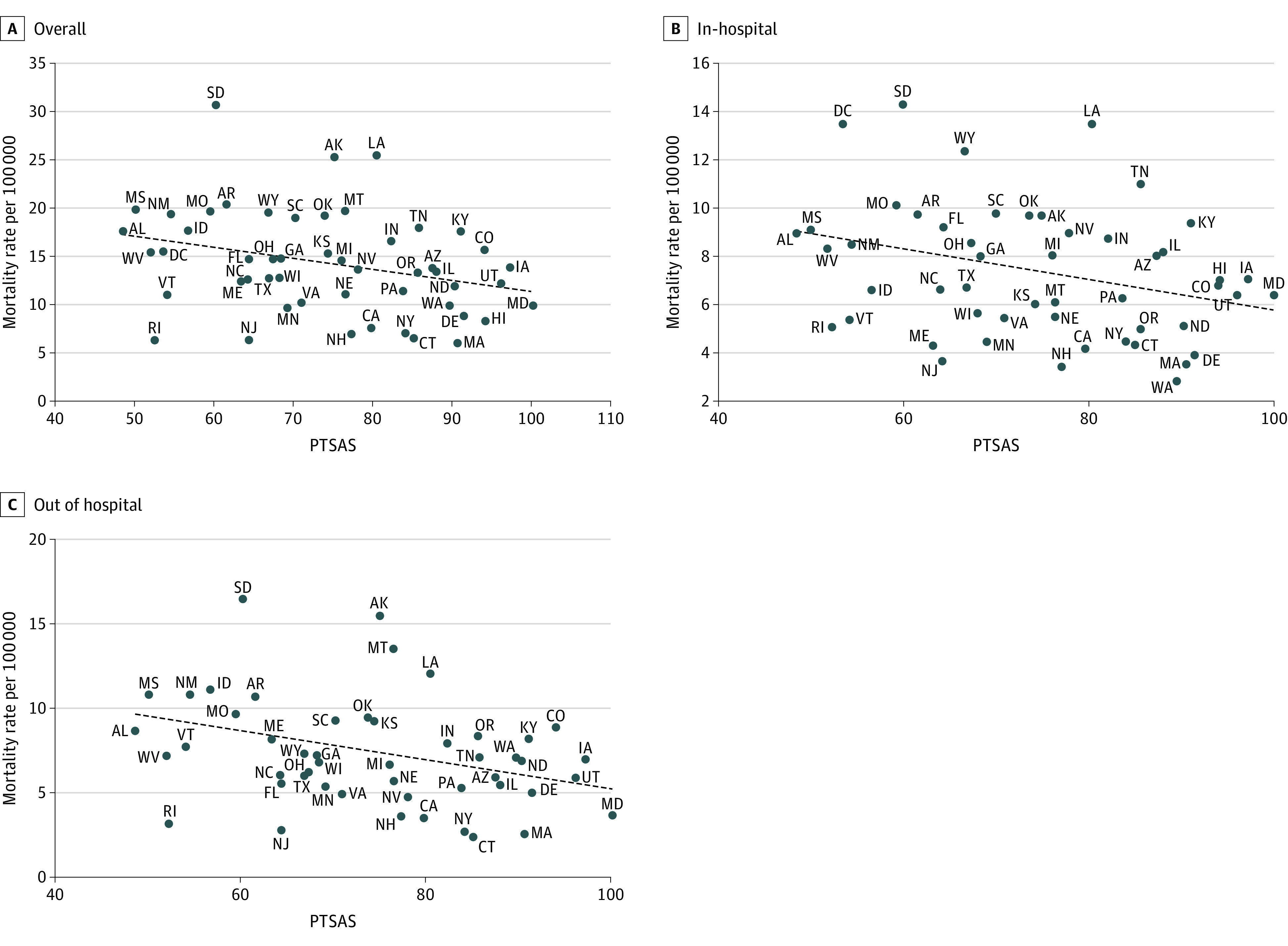
Mortality Rate by State and Pediatric Trauma System Assessment Score (PTSAS) A, Overall; B, in-hospital; and C, out-of-hospital mortality rate and PTSAS.

Using linear regression, each 1-point increase in the PTSAS was associated with 0.12 deaths (95% CI, −0.22 to −0.02) per 100 000 decrease in overall mortality (Table 3 and Figure 2). Increasing PTSAS was associated with a significant decrease in both in-hospital and out-of-hospital pediatric mortality (Table 3 and Figure 2).

**Table 3.** Validity Evidence for the Pediatric Trauma System Assessment Score

Mortality	Mean difference in mortality rate per 1-point increase in PTSAS (95% CI)	*P* value
Overall	−0.12 (−0.22 to −0.02)	.03
Out-of-hospital	−0.09 (−0.15 to −0.002)	.01
In-hospital	−0.06 (−0.12 to −0.01)	.02

Abbreviation: PTSAS, Pediatric Trauma System Assessment Score.

Many organizations have defined regions in the US developed for their own intents and purposes and these vary considerably. We used our scoring system to show regional scores in these differently defined regions (eFigure 2 in the Supplement).

## Discussion

The gap analysis in this cross-sectional study demonstrated significant variation between states in their resources and capability to care for injured children. The newly developed PTSAS, measuring the preparedness of a state pediatric trauma system, demonstrated significant association with improved pediatric mortality after traumatic injury in states with better scores. Several current regionalization models used by organizations that could be pertinent to pediatric trauma were used to show how resources may vary. Potentially, sharing of resources among regional states may improve the care of pediatric trauma patients in that region.

This project represents an objective evaluation of state pediatric trauma system development and readiness. It uses a consensus-based PTSAS tool by which hospitals, state agencies, and federal programs can assess and monitor their progress in establishing and improving capabilities to care for injured children, ultimately contributing to improvements in survival and functional outcomes after injury. Future use of the scoring system will allow a state to improve over time and promote alignment of pediatric emergency practice among neighboring states that may be leveraged in times of crisis. This is one approach to design systems of care to enable well-resourced states to work with underresourced states to improve care across state lines or within regions.

The goal of a state or regional trauma system is to appropriately identify and treat severely injured patients at specialized trauma centers and care for less-injured patients at lower-tier trauma centers. The system works in a coordinated effort to care for all ages of injured patients, without overwhelming specialized centers. For adults, development of trauma systems has been associated with decreased injury rates and mortality, but this has not consistently been demonstrated for children.^10,11,12,13,14^

We advocate for children’s interests to be recognized and integrated into trauma centers and system visions for the future as our country looks toward developing a strategic plan around a goal of zero preventable trauma deaths. The strategies for children should be developed by (1) considering the current state infrastructure to support pediatric trauma care; (2) defining parameters that are universally understood, have attainable and measurable answers, and have the potential to influence outcome; and (3) identifying which organizations are best positioned to assist with consensus building around pediatric trauma indicators that can be used to measure trauma system development in a state.

### Next Steps/Justification for State-Level Data

The current trauma system evaluation done by the ACS-COT does not include parameters for the evaluation of pediatric trauma care. Many parameters used in our scoring system are readily adaptable to an assessment of pediatric trauma capabilities in a state and could be used in a future scoring system. For rural and underserved environments, the most inexperienced care for children is often in the ranks of prehospital professionals and emergency departments. Telehealth and telesimulation as resources for clinician education, telemedicine for rural trauma care, and teleradiology to decrease repeat imaging will all have a future, enhanced role in pediatric trauma care and disaster preparedness.^15^ We will be unable to optimally care for injured children everywhere in the country, from the perspective of recognition of injuries, resuscitation, and transfer, unless we train front-line emergency care professionals. This can be accomplished as part of team building with trauma professionals, which includes improving education and the ability to use pediatric equipment.

Parallel to this project are the anticipated influences of the National Trauma Research Action Plan, and the Regional Medical Operations Center.^16,17^ The latter emerged during COVID-19 as an effective model already in existence and was promulgated through the efforts of the ACS-COT. Similarly, there is anticipated effort to develop a National Trauma and Emergency Preparedness System.^18^ As these interests gain traction, children must be included and integrated, and children’s health care professionals must be included in planning and development.

### Limitations

There were several limitations of this study. The qualitative data of the survey relied on the responsiveness of state officials. Self-reported data may be inaccurate if the individuals did not verify their responses. States with unfilled positions or new officials may have been disadvantaged with respect to knowledge or experience to complete the survey; some states varied in number of participating state officials, and both may have contributed to sampling bias.

This study spanned several years, and a few states now have verified pediatric trauma centers that did not exist when the project began. States may have improved parameters for pediatric trauma system evaluation within the intervening years that the study took place. Although the study group made every effort to obtain missing information from public documents, there were some states where this was not possible and could affect their overall score. The NPRP recently updated their state scores, and it was not possible to use these updated scores in our project. We expect that this parameter will change in the future as the ACS-COT will include pediatric readiness in trauma center verification beginning in 2023.^19^

## Conclusions

This cross-sectional study evaluated the landscape of state trauma system development for US children from the viewpoint of state and organizational leadership. The survey instrument encompassed the identification of key parameters in trauma system development that affect both children and adults but with a specific lens on how children are included. We developed a scoring system based on those parameters and found that states with a higher PTSAS had lower pediatric injury mortality. Mortality is not an ideal outcome in evaluating trauma systems, particularly in children, and including outcomes that assesses health care utilization, process of health outcomes such as care measures, functional outcomes (return to normal activities, play, sports, school, and mental PTSD) and quality of life, will be advantageous.^20^ This study can inform the integration of pediatric trauma parameters and scoring into the next version of the ACS trauma system scoring tool, emphasizing incremental change and progress over time. The current PTSAS is a starting point and can be modified over time but is a step toward assessing each state’s pediatric trauma care capabilities within defined regions of the country.

## Notes

Supplement. **eTable 1. **Survey Questions Sent to State Officials **eFigure 1. **Project Time line **eTable 2. **Delphi Committee Members **eTable 3. **Results From Delphi Scoring **eTable 4. **States Without Specific Pediatric Trauma System Assessment Score Parameter **eFigure 2. **Regionalization Schemas With Mean Pediatric Trauma System Assessment Score by Region
