# Supplementary material for: A Novel Approach to Assessment of US Pediatric Trauma System Development
Source: JAMA Surg. 2022 Sep 21;157(11):1042–9. doi: 10.1001/jamasurg.2022.4303 (PMC9494270; doi:10.1001/jamasurg.2022.4303)
Supplement: Supplement. — eTable 1. Survey Questions Sent to State Officials eFigure 1. Project Time line eTable 2. Delphi Committee Members eTable 3. Results From Delphi Scoring eTable 4. States Without Specific Pediatric Trauma System Assessment Score Parameter eFigure 2. Regionalization Schemas With Mean Pediatric Trauma System Assessment Score by Region [file jamasurg-e224303-s001.pdf]

Supplementary Online Content

Fallat ME, Treager C, Humphrey S, et al. A novel approach to assessment of US pediatric trauma system development. *JAMA Surg*. Published online September 21, 2022. doi:10.1001/jamasurg.2022.4303

eTable 1. Survey Questions Sent to State Officials

eFigure 1. Project Time line

eTable 2. Delphi Committee Members

eTable 3. Results From Delphi Scoring

eTable 4. States Without Specific Pediatric Trauma System Assessment Score Parameter

eFigure 2. Regionalization Schemas With Mean Pediatric Trauma System Assessment Score by Region

This supplementary material has been provided by the authors to give readers additional information about their work.

© 2022 Fallat ME et al. *JAMA Surgery.*

eTable 1. Survey Questions Sent to State Officials
(Italicized questions are from legacy data and could be updated by the state; Bold Questions are new survey questions developed for the study)

% of Population <10 Miles from High Level Pediatric Trauma Center.
% of Population 10–30 Miles from High Level Pediatric Trauma Center.
% of Population >30 Miles from High Level Pediatric Trauma Center.
% of Population <10 miles from High Level Adult or Pediatric Trauma Center.
% of Population 10–30 Miles from High Level Adult or Pediatric Trauma Center.
% of Population >30 Miles from High Level Adult or Pediatric Trauma Center.
% of Population <10 Miles from High-Mid Level Adult or Pediatric Trauma Center.
% of Population 10–30 Miles from High-Mid Level Adult or Pediatric Trauma Center.
% of Population >30 Miles from High-Mid Level Adult or Pediatric Trauma Center.
**Does the State have trauma system legislation?**
Where is your trauma office "administratively" located?
Does the State have a trauma system funding source(s)?
Does the State trauma system receive federal funds?
**Is there an annual budget for the trauma system?**
**Are any funds specifically for pediatric needs?**
Is there Trauma Program Accountability to State EMS Office (EMSO)?
**Does the State trauma system include pediatric needs (i.e. children are addressed in the state statute)?**
Does the State have enabling legislation to designate trauma centers?
**Does the State have legislation to designate pediatric trauma centers?**
Does the State have regulatory authority to limit the number of trauma centers?
Is there a state trauma plan available?
What is the basis for the State trauma plan?
Is there a Statewide Trauma Advisory Committee (TAC)?
**If yes, is there pediatric representation on the statewide TAC?**
Are there Regional TACs?
**If yes, is there Pediatric Representation on the Regional TAC?**
**Does the State promote/organize participation in pediatric injury prevention?**
Is the State trauma program involved in injury prevention efforts?
Is State trauma program involved in Public Information & Education (PI&E)(not related to injury prevention)?
**Does the state publicly report trauma registry data that includes pediatric trauma patients?**
**How is the State trauma data reported to the public?**
Is trauma included in the Statewide disaster plan?
**Does the State disaster plan include children?**
Does the State trauma program have its own Mass Casualty Incident (MCI) plan?

© 2022 Fallat ME et al. *JAMA Surgery.*

**Does the State trauma system legislation/plan include simulation and modeling for injured children?**
Is *there a State Disaster Triage Guideline?*
**Does the State hold mass casualty drills that include children?**
**If yes, how often?**
**Do hospitals within the state hold disaster drills that include children?**
**Do State disaster drills include surge planning for children?**
Are Trauma Center Levels designated by the State?
What is the method of Trauma Center designation/verification in the State?
Is there medical oversight for the State Trauma System?
Are CDC Field Triage Guidelines (2011) used in the State?
Is there a State Trauma Destination (Bypass) Protocol in place?
**Is there a State Pediatric Trauma Destination (Bypass) Protocol in place?**
**Do the State hospitals have transfer agreements for unavailable resources?**
Does the State have a Statewide PI Plan or Guide for Trauma?
**Are children's interests recognized in the Statewide PI Trauma plan?**
**Is there a State Trauma Registry (TR)?**
**If yes, is the TR used for Performance Improvement (PI)?**
**If yes, does State TR include Children?**
**Does the State have a separate pediatric report for trauma?**
Is the State TR electronically integrated with prehospital (EMS) data?
**Does the State EMS data include children?**
**Is the State EMS data used for pediatric PI?**
**What is the State Average Peds Ready Score for EDs that are Adult Trauma Centers?**
**What is the State Average Peds Ready Score for EDs that are Pediatric Trauma Centers?**
**What is the State average Pediatric Readiness (PR) Score for all EDs?**
**Do the State Adult Trauma Center EDs have guidelines for recognition of child abuse?**
**Is there State legislation for Child Fatality Review that is instructive on child abuse?**
**If yes, is there a mandatory death review of childhood deaths resulting from abuse?**
**Does the State have shaken baby parent education legislation?**
**If yes, give statute and year enacted?**
**Do State hospitals use ALARA (As Low As Reasonably Achievable) guidelines?**
**Do State Adult Trauma Centers use ALARA guidelines for CT use in children?**
**If no, please explain.**
**Are injured children typically worked up by the referring hospital before transfer?**
**Does the referring hospital discuss how to transfer a child?**
**Do State hospitals use telemedicine to communicate about pediatric trauma patients?**
**Does State have teleradiology sharing capability?**
**If yes, is it Statewide, system or hospital?**

© 2022 Fallat ME et al. *JAMA Surgery.*

**Does the State have access to pediatric inpatient burn care beds?**
**If yes, what are the resources for pediatric burn care?**
**Does the State have access to pediatric inpatient rehabilitation needs?**
**If yes, what are the resources?**
**Is the State rehab facility Commission on Accreditation of Rehabilitation Facilities (CARF) accredited for Peds?**
**Is the State rehab facility CARF accredited for adults?**
**Who directs the State rehab care?**
**Does the State outpatient rehabilitation model include pediatric trauma needs?**
**If yes, what are the resources?**
**Who directs the State outpatient rehab care?**
**Does the State offer ACS RTTDC courses?**

© 2022 Fallat ME et al. *JAMA Surgery.*

eFigure 1. Project Time line

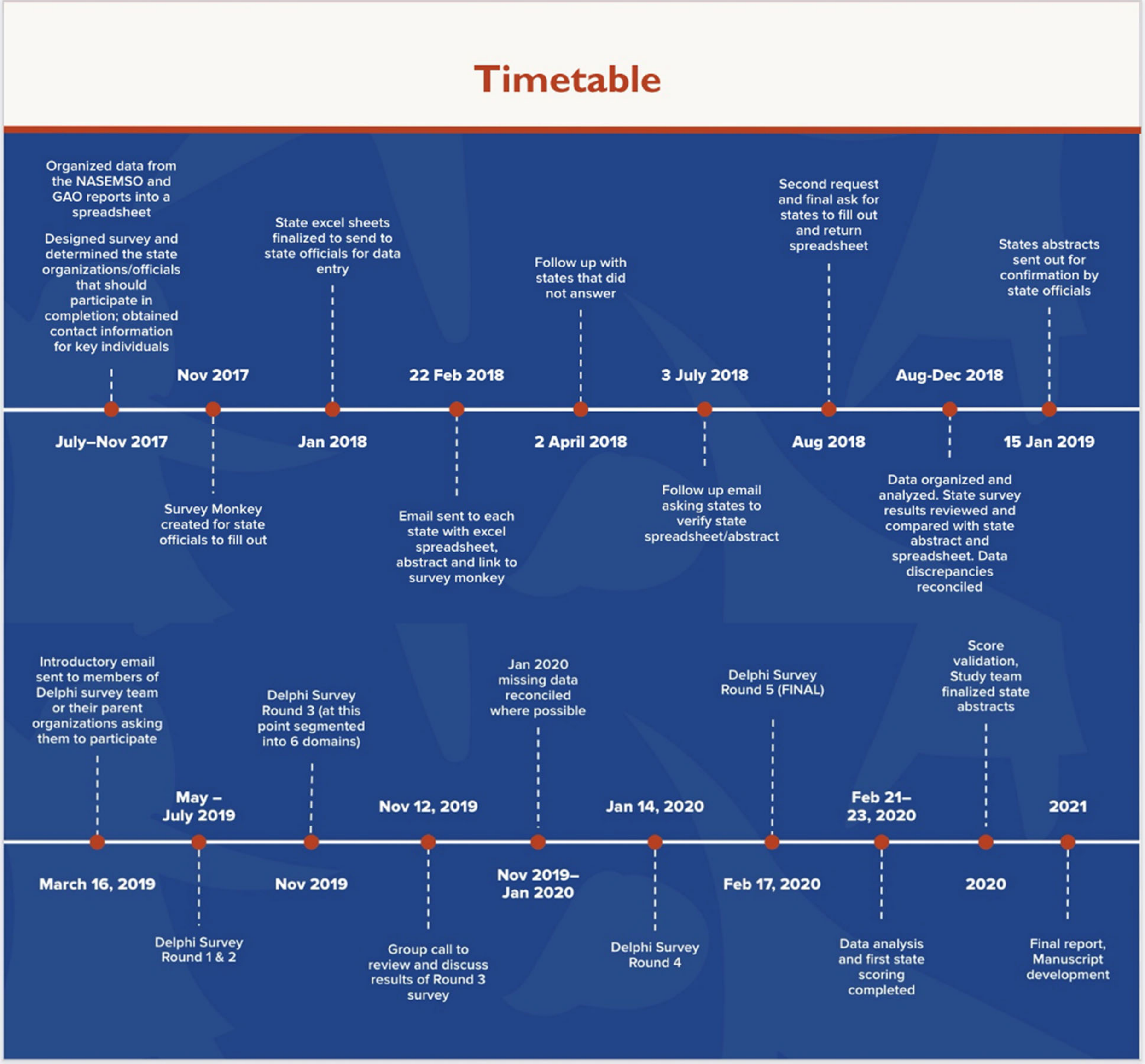

© 2022 Fallat ME et al. *JAMA Surgery.*

eTable 2. Delphi Committee Members
(*Designates a member of the leadership team)

Name	Title	Sector Representation
Amelia Collings, MD*	General surgery resident at University of Louisville, MWPSC clinical research fellow	Pediatric trauma research
Carrieann Drenton, MD	Practicing emergency medicine physician Sutter Medical Center, Sacramento, CA	Emergency medicine (non-academic setting)
Mary Fallat, MD*	Professor of surgery at University of Louisville	Pediatric trauma surgeon
Peter Fischer, MD, MS	Associate professor of surgery & anesthesia at University of Tennessee Health Science Center, Memphis, TN	Adult trauma & acute care surgeon; ACS-COT Representative; Geomapping
Tom Hartka, MD, MS	Assistant professor of emergency medicine at University of Virginia Health Sciences, Charlottesville, VA	Emergency medicine (academic setting); Medical examiner; Biomechanics and crash research
Tiffany Lightfoot, RN, MS	State Trauma Program Coordinator for Hawaii State Department of Health	Trauma Program Manager NASEMSO Representative
Bindi Naik Mathuria, MD, MPH	Associate professor of surgery & pediatrics at Baylor College of Medicine, Houston, TX	ACS-COT Future Trauma Leaders; Pediatric trauma surgeon
Terry Mullins, MPH, MBA	Chief of Arizona Department of Health Services, Bureau of EMS and Trauma	State EMS Director
Christian Niedzwecki, DO, MS	Associate professor of physical medicine & rehabilitation of Baylor College of Medicine, Houston, TX	Pediatric rehabilitation
Katherine Remick, MD	Associate professor pediatrics & surgery at Dell Medical School University of Texas, Austin, TX	Pediatric emergency medicine; EMS Director; NPRP
Frederick Rivara, MD, MPH*	Endowed chair of pediatric research in Department of pediatrics at the University of Washington, Seattle, WA	Pediatric and trauma research
Frederick Rogers, MD, MS, MA*	Professor of surgery at University of Pennsylvania, Philadelphia, PA	Rural trauma surgeon and trauma research
Morgan Scaggs, BHS, NRP	Kentucky EMSC Project director	EMSC representative
Joey Scollan, DO	NASEMSO medical director council representative	State EMS medical director
Mary Wethington, MSN, RN, CEN, CPEN	Staff nurse at McKenna David Pediatric Emergency Center, instructor at University of Kentucky College of Nursing	Emergency Nurses Association representative, pediatric nurse

© 2022 Fallat ME et al. *JAMA Surgery.*

eTable 3. Results From Delphi Scoring

Variable	Round 1 Weighted Average	Round 2 Weighted Average	Domain or Reason for Removal	Round 4 Weighted Average	Round 5 Inclusion Consensus
Q1. Percentage of pediatric patients that live within 30mi of a high level (I or II) pediatric trauma center (data comes from GAO report)	7.43	6.6	The panel felt that access to any trauma center (adult or pediatric) was more important, and feasible, than access to specifically a high-level pediatric trauma center	-	-
Q2. The US GAO has provided a national report by state showing the percentages of children living in proximity to a PTC based on quartiles. The exact percentage of children living in proximity to a PTC should carry more or less weight based on quartiles (i.e. 0-24.9%, 25-49.9%, etc). Q2	7.36	6.58	The expert panel felt that the exact percentage was more important than percentiles	-	-
Q3. Percentage of pediatric patients that live within 30mi of either a high level (I or II) pediatric or adult trauma center Q3	7.64	7.46	***Access to Care***	9.08	Yes
Q4. The US GAO has provided a national report by state showing the percentages of children living in proximity to either a high level (I or II) adult or PTC based on quartiles. The exact percentage of children living in proximity to either a high level (I or II) adult or PTC should carry more or less weight based on quartiles (i.e. 0-24.9%, 25-49.9%, etc).	6.36	7.23	The expert panel felt that the exact percentage was more important than percentiles	-	-
Q5. Percentage of pediatric patients that live within 30mi of either a high or mid-level (I, II, or III) pediatric or adult trauma center (i.e. access to any Level I-III Adult or Pediatric Trauma Center).	7.29	6.92	***Access to Care***	9.08	Yes
Q6. The US GAO has provided a national report by state showing the percentages of children living in proximity to either a high or	6.93	6.85	The expert panel felt that the exact percentage	-	-

© 2022 Fallat ME et al. *JAMA Surgery.*

mid-level (I, II, or III) adult or PTC based on quartiles. The exact percentage of children living in proximity to either a high or mid-level (I, II, or III) adult or PTC should carry more or less weight based on quartiles (i.e. 0-24.9%, 25-49.9%, etc). https://i.imgur.com/VB5Zi32.png			was more important than percentiles		
Q7. There is state legislation for trauma system development.	9	8.86	***Legislation and Funding***	8.15	Yes
Q8. Legislation, if present, specifically addresses injured children and includes rules regarding the specific needs of injured children - i.e., staff education, equipment	8.42	7.07	The panel felt that pediatric needs would be addressed in legislation or by satisfying the other metrics in the Legislation Domain	-	-
Q9. State agencies, health department, or the trauma system participate in organized injury prevention efforts for children.	8.93	7.5	***Injury Prevention and Recognition***	8.08	Yes
Q10. State disaster plan includes children	9.36	9.64	***Disaster***	8.77	Yes
Q11. State holds mass casualty drills that include children	7.79	7.64	***Disaster***	8.77	Yes
Q12. Mass casualty drills include facilities planning for transfer of children to accommodate surge	8.5	8.86	***Disaster***	8.77	Yes
Q13. Mass casualty drills include both a process for identifying children to be moved and verifying facilities receiving children as having appropriate resources to provide optimal care	8.14	8.93	***Disaster***	8.77	Yes
Q14. State trauma system planning, simulation, and modeling includes children	8.79	8.5	***Legislation and Funding***	8.15	Yes
Q15. State has enabling legislation to designate pediatric trauma centers	7.36	8.36	***Legislation and Funding***	8.15	Yes
Q16. State has an EMS patient triage or destination determination protocol (e.g., Guidelines for Field Triage of Injured Patients) for injured children (nearest hospital versus appropriate trauma center)	8.14	8	***Access to Care***	9.08	Yes
Q17. State-based trauma registry (TR) includes data on pediatric trauma patients	9.85	9.21	The expert panel felt that by publicly reporting	-	-

© 2022 Fallat ME et al. *JAMA Surgery.*

			trauma data that includes children (Q21), this metric would be satisfied and thus redundant.		
Q18. Trauma Registry data in the state is used for children's performance improvement (PI) and is evaluated separately from adults	7.79	8.14	***Quality Improvement and Trauma Registry***	8.77	Yes
Q19. A fraction of statewide budgeted funds is specifically dedicated to pediatric needs or interests	7	7.57	***Legislation and Funding***	8.15	Yes
Q20. There is mandatory pediatric representation on your state Trauma Advisory Council	8.57	8.93	***Legislation and Funding***	8.15	Yes
Q21. State publicly reports trauma data that includes children	8.43	8.21	***Quality Improvement and Trauma Registry***	8.77	Yes
Q22. State pediatric EMS data is used for EMS service or system PI and is evaluated separately from adults	7.29	8.07	***Quality Improvement and Trauma Registry***	8.77	Yes
Q23. Hospitals in the state, in general, use as low as reasonably achievable (ALARA) guidelines for radiographic imaging	7.36	7.43	***Pediatric Readiness***	8.77	Yes
Q24. All levels of trauma center (adult, pediatric, or mixed) have education programs for their staff that include recognition of child abuse	8.43	8.5	***Injury Prevention and Recognition***	8.08	Yes
Q25. State legislation is in place to review all child fatalities due to injury, including child abuse	8	8.29	***Injury Prevention and Recognition***	8.08	Yes
Q26. Some hospitals in the state use telemedicine for communication with pediatric trauma centers including review of patient charts and images to determine the need for and assistance with transfer.	6.6	6.5	The weighted average fell below the set threshold of 7.0 and thus was eliminated	-	-
Q27. State has burn beds available for children	7.29	6.93	***Access to Care***	9.08	Yes
Q28. State has inpatient rehabilitation beds available for children under 14 years old in a	7.21	7.57	***Access to Care***	9.08	Yes

© 2022 Fallat ME et al. *JAMA Surgery.*

pediatric rehabilitation unit (the unit can be within a rehabilitation facility but is specifically designated for children).					
Q29. Rehabilitation facilities utilize CARF (Commission on Accreditation of Rehabilitation Facilities) accreditation for pediatric rehabilitation	6.79	6.5	The weighted average fell below the set threshold of 7.0 and thus was eliminated	-	-
Q30. State requires transfer guidelines and defined processes/protocols be in place at each hospital	7.71	8.29	***Pediatric Readiness***	8.77	Yes

© 2022 Fallat ME et al. *JAMA Surgery.*

eTable 4. States Without Specific Pediatric Trauma System Assessment Score (PTSAS) Parameter

Domain	Parameter	States not in compliance with PTSAS parameter
Disaster	State disaster plan includes children	AL, FL, ME, MI, MN, MS, MO, MT, NH, NJ, NC, OH, SC, TX, WV
	State holds mass casualty drills that include children	AL, FL, MI, NC, SC, VA, WV
	Mass casualty drills include facilities planning for transfer of children to accommodate surge	AL, CA, FL, GA, ID, KS, ME, MS, MO, NE, NV, NH, NJ, NY, OH, OR, PA, SC, VA, WV, WY
	Mass casualty drills include both a process for identifying children to be moved and verifying facilities receiving children as having appropriate resources to provide optimal care.	-
Legislation and Funding	There is state legislation for trauma system development	VT, DC
	There is mandatory pediatric representation on your state Trauma Advisory Council	AL, AZ, AR, GA, ID, IL, KS, MA, MI, MS, MO, NV, NJ, OK, RI, SD, VT
	State trauma legislation specifically addresses injured children and includes planning, simulation, and modeling	AL, CA, CO, FL, ID, ME, MN, MO, MT, NM, OR, PA, RI, SC, SD, VT, WV, WI, WY
	State has enabling legislation to designate pediatric trauma centers	AL, CA, KS, ME, MT, NM, NC, ND, OK, OR, RI, SD, TN, TX, VT, WV, WY, DC
	There are state funds designated for pediatric trauma care	AL, CO, DE, FL, GA, ID, IL, IN, IA, LA, MN, MS, MO, NV, NH, NJ, NM, NY, NC, ND, OH, OK, RI, SC, SD, TX, VT, WV, MI, WY, DC
Access to Care	State has an EMS patient triage or destination determination protocol (e.g., Guidelines for Field Triage of Injured Patients) for injured children (nearest hospital versus appropriate trauma center)	AL, AK, AZ, AR, CA, GA, ID, IN, KS, KY, ME,MN, MT, NE,NC, ND, OH, OR, RI, SD,UT, VT, VA,WI, WY, DC
	State has access to inpatient rehabilitation beds available for children under 14 years old in a pediatric rehabilitation unit (the unit can be within a rehabilitation facility but is specifically designated for children).	AK, ID, RI, WY
	State has access to burn beds available for children	CT, MS, NE, NJ, NY, PA, SD, VT, WV, WY

© 2022 Fallat ME et al. *JAMA Surgery.*

	Percentage of pediatric patients that live within 30mi of either a high level (I or II) pediatric or adult trauma center	AK, AR, MT, NM, SD, WY
	Percentage of pediatric patients that live within 30mi of either a high or mid-level (I, II, or III) pediatric or adult trauma center	AK
Injury Prevention and Recognition	State legislation is in place to review all child fatalities due to injury, including child abuse	ID, MI, MN, MS, MO, NH, NM, NY, RI, WI
	All levels of trauma center (adult, pediatric, or mixed) have education programs for their staff that include recognition of child abuse	AZ, AR, MN, NM, WA, WV, WI, DC
	State agencies, health department, or the trauma system lead efforts in organized injury prevention for children	MS
Quality Improvement and Trauma Registry	Summary data from state-based trauma registry is publicly reported and includes pediatric trauma patients	AR, DE, FL, GA, ID, LA, ME, MI, NJ, NM, NC, RI, SD, TX, VT, VA, WV, DC
	Trauma Registry data in the state is used for children's performance improvement (PI) and is evaluated separately from adults	AL, AR, CA, ID, IL, IN, ME, MS, MT, NE, NJ, NM, NC, OK, RI, TX, VT, VA, WV, WI, WY, DC
	State pediatric EMS data is used for EMS service or system PI and is evaluated separately from adults	AL, AK, CT, GA, ID, KS, LA, NV, NJ, PA, SC, SD, TN, VA, WI, DC
Pediatric Readiness	The state measures pediatric readiness of its emergency departments	AL, CT, FL, GA, HI, ME, MA, MS, MO, NV, NM, NC, OH, OK, TN, TX
	State requires transfer guidelines and defined processes/protocols be in place at each hospital	AK, AR, KS, LA, MN, MO, NE, NH, OH, RI, SC, SD, VT, WV, DC
	Hospitals in the state, in general, use as low as reasonably achievable (ALARA) guidelines for radiographic imaging	AL, FL, IN, KY, MI, MS, MO, NM, OH, OK, RI, TX, VT, WA, DC

© 2022 Fallat ME et al. *JAMA Surgery.*

eFigure 2. Regionalization Schemas With Mean Pediatric Trauma System Assessment Score (PTSAS) by Region
a) Emergency Medical Services for Children (EMSC); **b)** American College of Surgeons Committee on Trauma (ACS-COT); **c)** National Association of State EMS Officials (NASEMSO); **d)** U.S. Census Bureau; **e)** American Burn Association (ABA)

a)

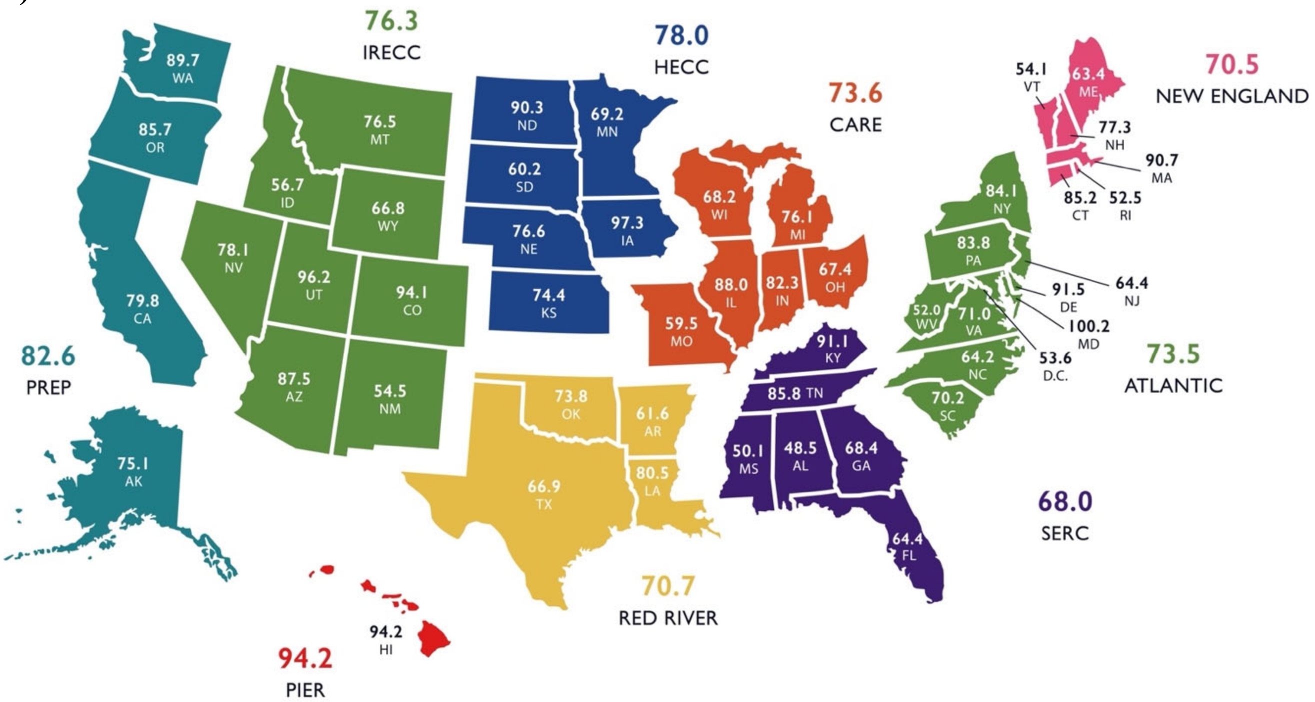

b)

© 2022 Fallat ME et al. *JAMA Surgery.*

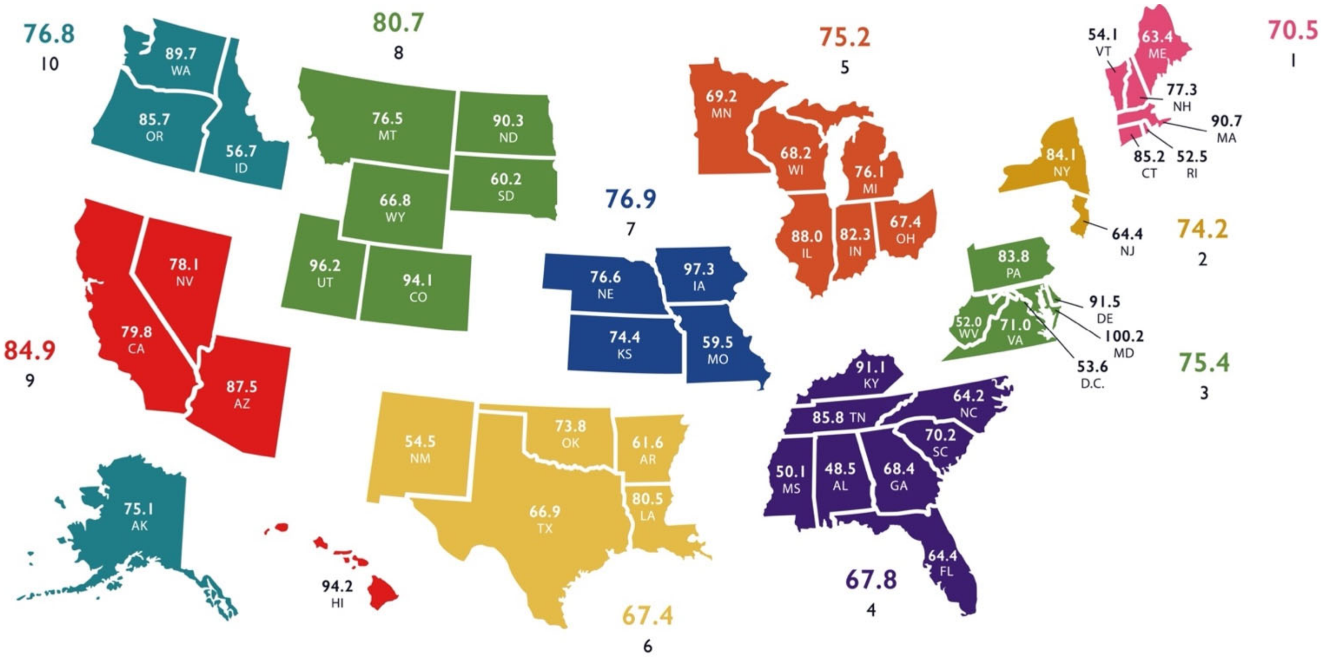

c)

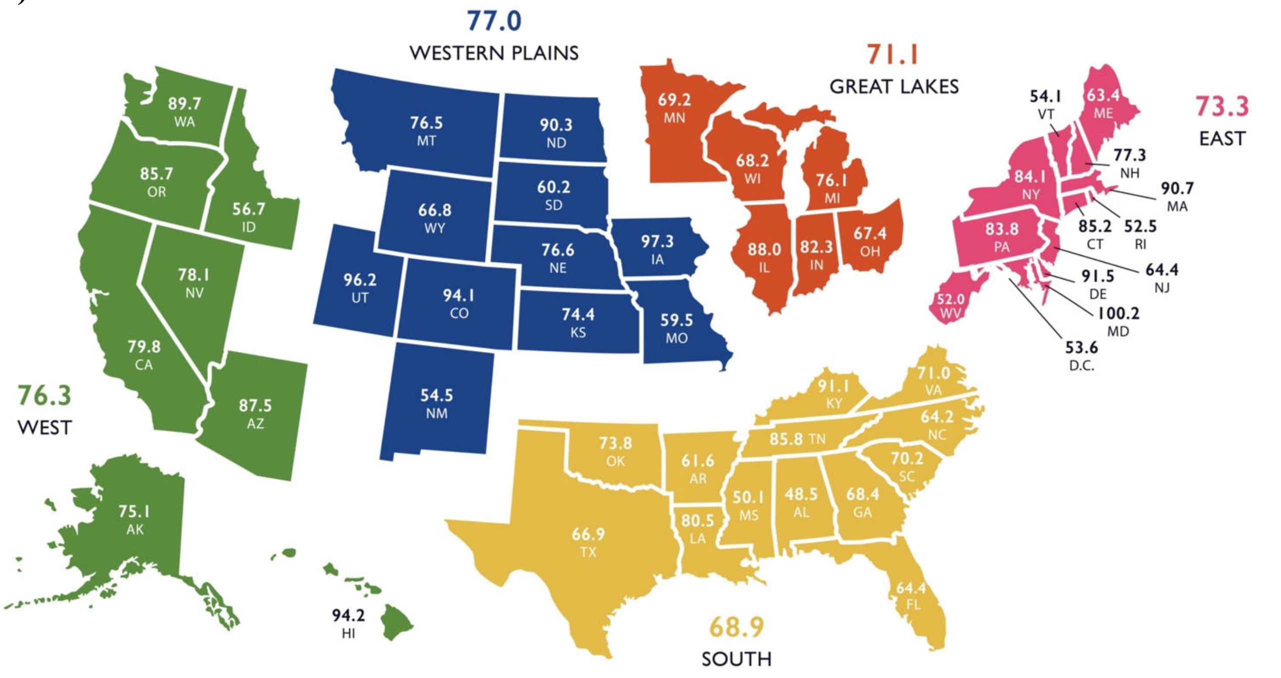

d)

© 2022 Fallat ME et al. *JAMA Surgery.*

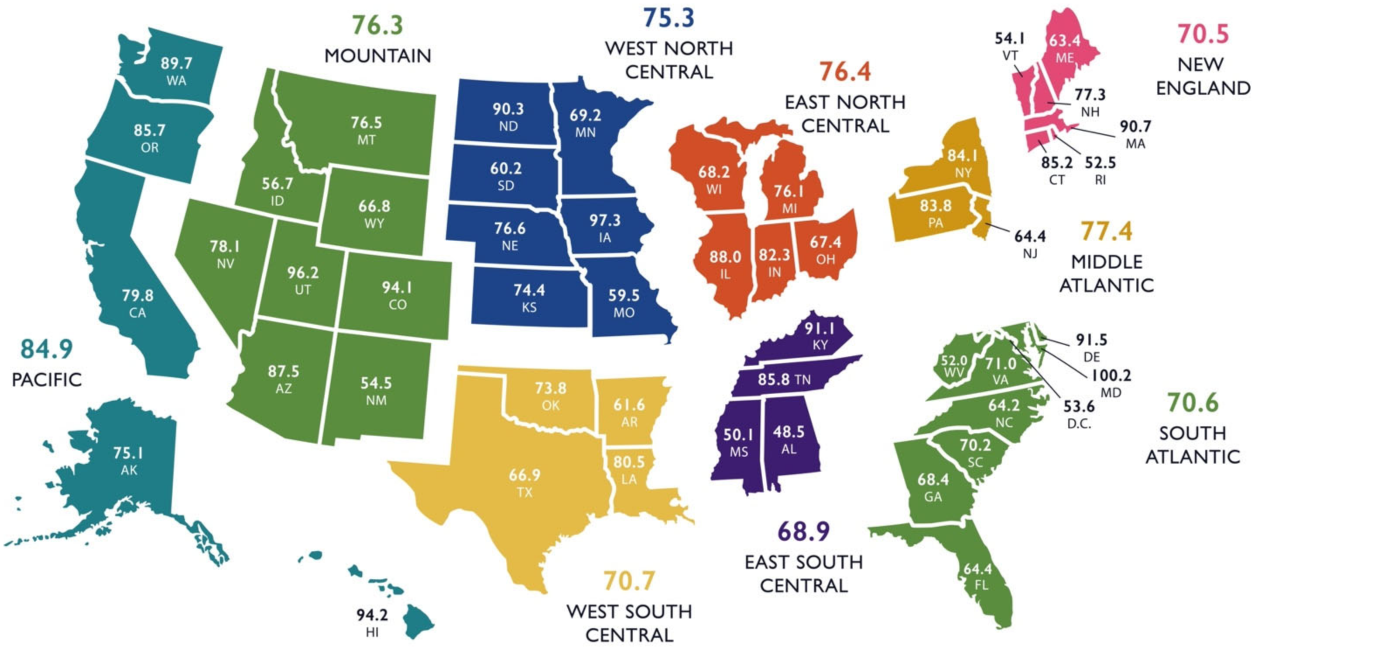

e)

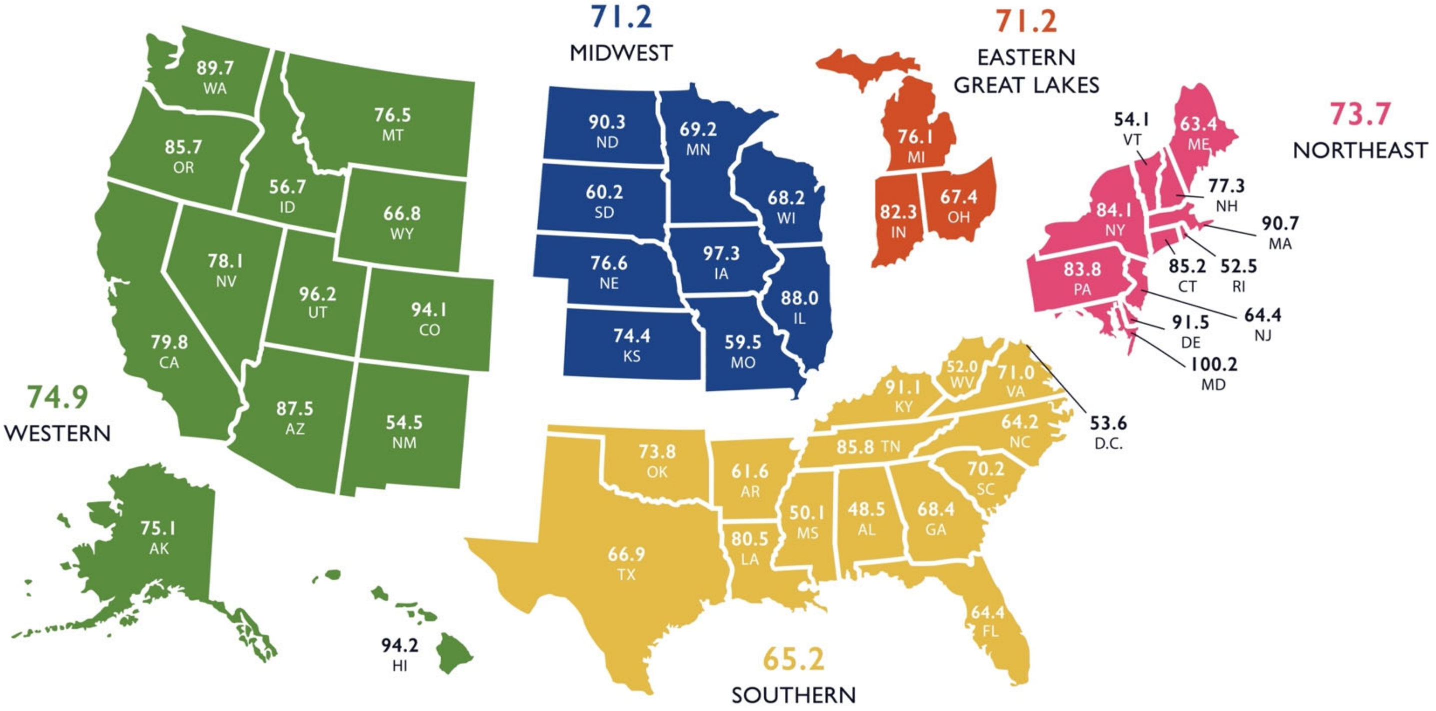

© 2022 Fallat ME et al. *JAMA Surgery.*
